# A transcriptional complex composed of ER(α), GATA3, FOXA1 and ELL3 regulates *IL-20* expression in breast cancer cells

**DOI:** 10.18632/oncotarget.17459

**Published:** 2017-04-27

**Authors:** Jae Yong Lee, Young Joon Park, Nuri Oh, Kyu Bum Kwack, Kyung-Soon Park

**Affiliations:** ^1^ Department of Biomedical Science, College of Life Science, CHA University, Seoul, Korea

**Keywords:** estrogen receptor alpha, GATA3, FOXA1, ELL3, interleukin-20

## Abstract

Interleukin-20 (*IL-20*) is a member of the IL-10 family. *IL-20* expression is regulated by a transcription elongation factor, Ell3, in estrogen receptor-positive (ER(+)) breast cancer cells. In this study, we demonstrated that ER(α), GATA3 and FOXA1 form a transcriptional complex with Ell3 to regulate *IL-20* expression in ER(+) breast cancer cells. We also determined that GATA3 and FOXA1 share a binding site with ER(α) in the interleukin-20 promoter. Furthermore, we found that FOXA1 represses *IL-20* expression, whereas GATA3 and ER(α) activate it. In addition, we demonstrated that Ell3 associates with ER(α) to increase its binding affinity to the *IL-20* promoter, which may prevent FOXA1 binding to the same region of this promoter. Our results expand upon the current understanding of the regulatory mechanism of *IL-20* in cancer.

## INTRODUCTION

Estrogen receptor alpha ER(α) is a critical nuclear hormone receptor that regulates breast epithelial cell proliferation and apoptosis. It also contributes to the malignancy of breast cells, and approximately 75% of breast cancers have been reported to be estrogen receptor positive ER(+). Therefore, the ER signaling pathway has been studied as a therapeutic target in breast cancer therapy. ER(+) breast cancer patients are typically treated with endocrine-based therapy; however, resistance to endocrine-based drugs is an emerging challenge. Endocrine therapy resistance is considered to result from the complex crosstalk between estrogen signaling and other oncogenic signaling pathways. In response to estrogen, ER(α) coordinates with coactivators and corepressors to induce a physiological response, and deregulation of coregulatory mechanisms is associated with the development and metastasis of breast cancer [1, 2].

Thus, a thorough understanding of the coordinating partners and transcriptional target genes of ER(α) is critical to identify novel therapeutic targets and to overcome endocrine therapy resistance in breast cancer.

Ell3 was originally identified as a testis-specific transcription elongation factor belonging to the eleven-nineteen lysine-rich leukemia (Ell) family, but its nucleotide sequence is divergent from those of Ell1 and Ell2 [3]. In mouse embryonic stem cells (mESCs), Ell3 associates with enhancers and mediates the occupancy of RNA Pol II at the promoter-proximal regions of development-related genes to prime subsequent gene activation following differentiation signals by recruiting the super elongation complex to these loci [4]. In addition, Ell3 promotes p53 degradation in mESCs, which contributes to the protection of differentiating cells [5]. We recently reported that ectopic expression of Ell3 stabilizes p53 in an ER(+) breast cancer cell line by activating the expression of interleukin 20 (*IL-20*), leading to chemosensitization of MCF7 cells upon CDDP treatment [6].

*IL-20* is a pleiotropic cytokine that is associated with inflammatory diseases, such as rheumatoid arthritis and atherosclerosis [7]. Recently, several studies have demonstrated that *IL-20* plays a critical role in tumor progression. *IL-20* promotes the migration and invasion of bladder cancer cells by activating ERK pathway-mediated MMP-9 protein expression [8]. Furthermore, *IL-20* activates STAT3 and ERK signaling in oral cancer cells to increase cell proliferation and colony formation [9]. *IL-20* expression is induced by direct binding of the NF-kB p50/p65 heterodimer in human keratinocytes [10]. Although *IL-20* is assumed to be a key cytokine in the pathogenesis of cancer, little is known about the factors involved in its regulation in cancer.

In this study, we show that Ell3, ER(α), GATA3 and FOXA1 form a transcriptional complex to regulate *IL-20* expression in ER(+) breast cancer cell lines. We further demonstrate that Ell3 associates with ERα and GATA3 to enhance binding affinity to the *IL-20* promoter. We also show that FOXA1 functions as a transcriptional repressor of *IL-20* by interfering with binding of ER(α) and GATA3 to the *IL-20* promoter.

## RESULTS

### ER(α) regulates IL-20 expression in breast cancer cells

In a previous study, we reported that transcription elongation factor Ell3 directly regulates *IL-20* expression in an ER(+) breast cancer cell line, MCF7, but not in ER(−) breast cancer cell lines [6]. Based on these results, we hypothesized that ER(α) is a transcriptional activator of *IL-20* expression in MCF7 cells.

**Figure 1:**
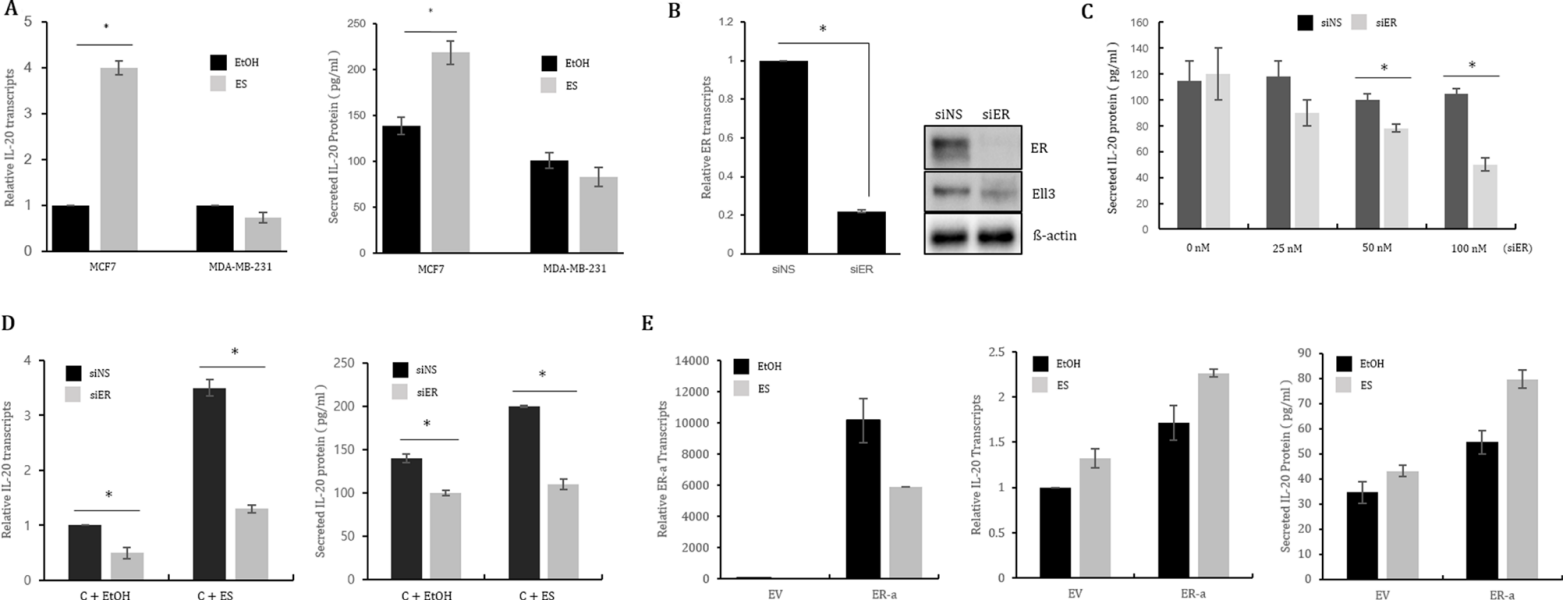
ER(α) regulates *IL-20* expression in MCF7 cells (**A**) The effect of estrogen treatment on *IL-20* expression. *IL-20* expression following ES treatment was analyzed by real-time RT-PCR and ELISA in MCF7 and MDA-MB-231 cells treated with ES for 48 h. (**B**) The suppression efficiency of siRNA targeting ER(α) was analyzed by real-time RT-PCR and immunoblot assay in MCF7 cells transfected with 100 nM of siRNA for 48 h. (**C**) The effect of ER(α) suppression on *IL-20* expression. The amount of secreted *IL-20* protein was analyzed by ELISA for MCF7 cells transfected with the indicated concentrations of siRNA for 48 h. (**D**) The effect of estrogen treatment on *IL-20* expression in MCF7 cells transfected with siER(α). MCF7 cells were transfected with siNS or siER(α) for 24 h, followed by treatment with ES for 48 h, and they were then analyzed by real-time RT-PCR and ELISA. (**E**) The effect of ER(α) overexpression on *IL-20* expression in the presence or absence of ES. ER(α) transcript, *IL20* transcript and secreted *IL-20* protein levels were analyzed in MCF7 cells transfected with a control (EV) or ER(α) expression plasmid (ER-α) for 48 h. Abbreviation: ES, estrogen 10 nM; EtOH (0.1%), ethanol; siNS, non-specific siRNA; siER, siRNA targeting ER(α); ER, estrogen receptor alpha. The error bars represent the standard errors from three independent experiments, which were each performed using triplicate samples. *P < 0.05 (Student's *t*-test).

As a first step in evaluating whether ER(α) activity is associated with *IL-20* expression, we examined the effect of estrogen on *IL-20* expression in ER(+) MCF7 cells and ER(−) MDA-MB-231 cells. Because ER(α) is translocated into the nucleus to function as a transcriptional activator following estrogen treatment, we hypothesized that *IL-20* expression in ER(+) cells would be enhanced by estrogen if its expression is regulated by ER(α). As expected, the *IL-20* RNA and secreted protein levels were significantly increased by estrogen in MCF7 cells but did not change in MDA-MB-231 cells (Figure 1A). To examine the effect of ER depletion on *IL-20* expression in MCF7 cells following estrogen treatment, we first tested the suppression efficiency of siRNA targeting ER(α). As shown in Figure 1B, 100 nM siRNA decreased ER(α) expression by up to 70% at both the RNA and protein levels. Following transfection of MCF7 cells with 25 nM, 50 nM or 100 nM siER(α), the amount of secreted *IL-20* decreased in proportion to the concentration of siRNA targeting ER(α) (Figure 1C). Furthermore, depletion of ER(α) by siRNA impeded the effect of estrogen on enhancing *IL-20* expression (Figure 1D). These results indicate that ER(α) functions to activate *IL-20* transcription in ER(+) MCF7 cells. To our surprise, the ectopic expression of ER(α) in MDA-MB-231 cells enhanced the expression of *IL-20* at both the RNA and protein levels by approximately ~2-fold in the presence or absence of estrogen, indicating that ER(α) activated *IL-20* expression in the triple negative breast cancer cell line (Figure 1E). Consistent with the results for MCF7 cells, *IL-20* expression in another ER(+) breast cancer cell line, T47D, was also enhanced by estrogen treatment and suppressed by the depletion of ER(α) (Supplementary Figure 2A–2C).

### ER(α) collaborates with Ell3 to regulate IL20 expression

**Figure 2:**
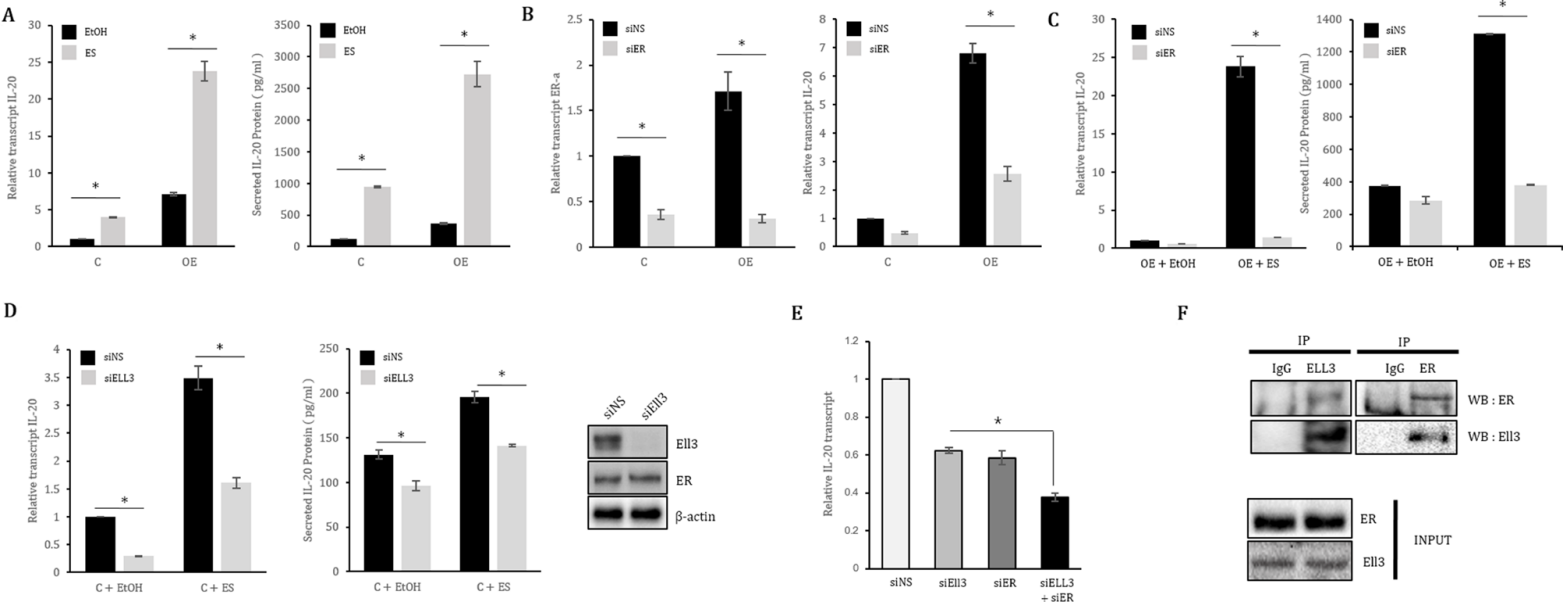
ER(α) collaborates with Ell3 to regulate *IL-20* expression in MCF7 cells (**A**) The effect of estrogen treatment on *IL-20* expression in Ell3-overexpressing MCF7 (Ell3 OE) cells. Control and Ell3 OE cells were treated with ES for 48 h, and then *IL-20* expression was analyzed by real-time RT-PCR and ELISA. (**B**) The effect of ER(α) suppression on *IL-20* expression in Ell3 OE cells. Ell3 OE cells were transfected with siNS or siER(α) for 48 h, followed by analysis by real-time RT-PCR and ELISA. (**C**) The effect of ES treatment on *IL-20* expression in ER(α)-suppressed Ell3 OE cells. Ell3 OE cells were transfected with siNS or siER(α) for 24 h, followed by treatment with ES for 24 h and analysis by real-time RT-PCR and ELISA. (**D**) The effect of ES treatment on *IL-20* expression in Ell3-suppressed MCF7 control cells. The suppression efficiency of siRNA targeting Ell3 was analyzed by immunoblot assay in MCF7 cells transfected with siRNA for 48 h (left panel). Control cells were transfected with siNS or siEll3 for 24 h and were then treated with ES for 24 h, followed by analysis of *IL-20* expression by real-time RT-PCR and ELISA (middle and right panels). (**E**) The effect of co-suppression of Ell3 and ER(α) on *IL-20* expression in MCF7 cells. (**F**) Co-immunoprecipitation (IP) between ER(α) and Ell3. MCF7 cell lysates were precipitated with anti-ER(α) or anti-Ell3 antibody-conjugated agarose and blotted with either an anti-Ell3 or anti-ER(α) antibody as indicated. Abbreviation: C, control MCF7 cells; OE, Ell3-overexpressing cells; ES, estrogen (10 nM); EtOH (0.1%), ethanol; siNS, non-specific siRNA; siER, siRNA targeting ER(α); siEll3, siRNA targeting Ell3; ER, estrogen receptor (α); IP, immunoprecipitation; WB, western blot. The error bars represent the standard errors from three independent experiments, which were each performed using triplicate samples. *P < 0.05 (Student's *t*-test).

To determine whether the Ell3-mediated enhancement of *IL-20* transcriptional expression is associated with ER(α), we examined the effect of estrogen on *IL-20* expression in Ell3 OE cells. *IL-20* expression was enhanced by Ell3 overexpression and was further increased by estrogen treatment (Figure 2A). Depletion of ER(α) resulted in a decrease in *IL-20* expression in Ell3 OE cells and in the control MCF7 cells (Figure 2B). Furthermore, the effect of estrogen on enhancing *IL-20* expression was impeded by siER(α) in Ell3 OE cells (Figure 2C). Following suppression of Ell3 by siRNA in MCF7 cells, *IL-20* expression was decreased, and the effect of estrogen on enhancing *IL-20* expression was inhibited (Figure 2D). Similar to the results for MCF7 cells, the suppression of Ell3 resulted in a decrease in *IL-20* expression in both the presence and absence of estrogen (Supplementary Figure 2D–2E). Cotransfection of siER(α) and siEll3 into MCF7 cells had an additive effect on suppressing *IL-20* expression compared with the transfection of siER(α) or siEll3 alone (Figure 2E). We next performed a co-immunoprecipitation experiment to determine whether the transcription elongation factor Ell3 directly associates with the transcription factor ER(α) to regulate *IL-20* expression. Following immunoprecipitation of total cell extracts of MCF7 cells with an anti-ER(α) or anti-Ell3 antibody, ER(α) or Ell3 was co-immunoprecipitated, respectively (Figure 2F). Taken together, these results demonstrate that Ell3 and ER(α) form a transcriptional complex to activate *IL-20* expression.

### ER(α) directly binds to the IL-20 and Ell3 promoters to regulate their gene expression

**Figure 3:**
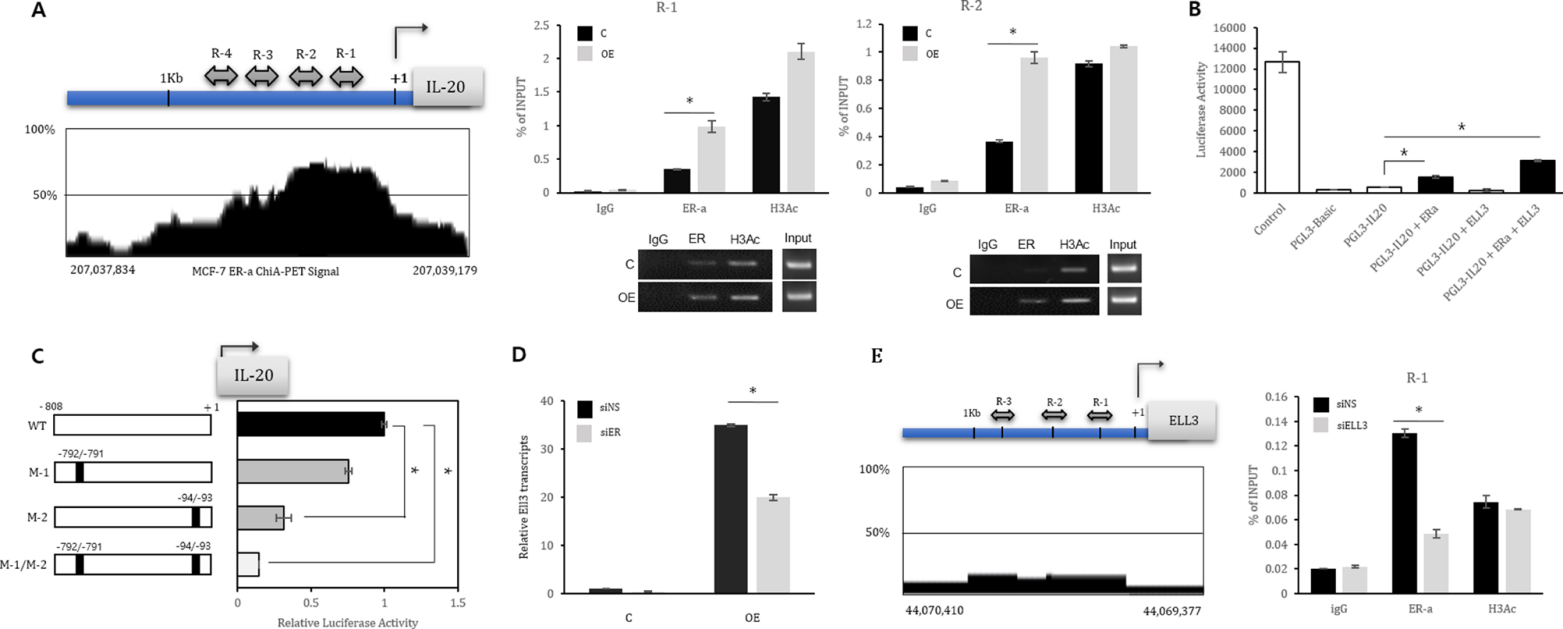
ER(α) is a transcriptional activator of *IL-20* and Ell3 (**A**) Left panel: Schematic diagram of the *IL-20* promoter. The *IL-20* promoter was divided into four regions based on the Paired-End Tag Sequencing (ChIA-PET) signal, named R1 to R4 (R1, chr 206,863,966 ~ 206,864,160; R2, chr 206,864,125 ~ 206,864,322; R3, chr 206,864,355 ~ 206,864,571; and R4, chr 206,864,727 ~ 206,864,950). Right panel: Chromatin from control and Ell3 OE cells was immunoprecipitated (ChIP) with antibodies against IgG, ER(α) and H3Ac. The PCR results for R1 and R2 of the *IL-20* promoter are presented. (**B**) Luciferase activity assay with the *IL-20* promoter. 293T cells were transfected with the indicated plasmid for 24 h, and cell lysates were analyzed for luciferase activity. The positive control is the pGL3-control plasmid, and the negative control is the pGL3-Basic plasmid. (**C**) Luciferase activity assay with the mutated *IL-20* promoter. ERE half sites on the *IL-20* promoter were point mutated as described in the Material & Methods and Figure S1. 293T cells were cotransfected with the ER(α) plasmid and the indicated plasmid for 24 h, and cell lysates were analyzed for luciferase activity. WT is the pGL3-IL20 plasmid. M-1 is the pGL3-IL20 plasmid with mutation of the -791 and -792 sequences. M-2 is the pGL3-IL20 plasmid with mutation of the -94 and -95 sequences. M-1/M-2 is the pGL3-IL20 plasmid with a double mutation of the -791 ~ -792 and -93 ~ -94 sequences. (**D**) The effect of ER(α) suppression on Ell3 expression. MCF7 cells were transfected with siNS or siER(α) for 48 h, and then Ell3 expression was analyzed by real-time RT-PCR. (**E**) Left panel: Schematic diagram of the Ell3 promoter. The regions containing ER(α) consensus binding sequences (EREs) are indicated as R1 to R3 (R1, chr 43,777,200 ~ 43,777,301; R2, chr 43,777,402 ~ 43,777,551; and R3, chr 43,777,886 ~ 43,778,042). Right panel: Chromatin from MCF7 cells transfected with siNS or siEll3 was immunoprecipitated (ChIP) with antibodies against IgG, ER(α) and H3Ac. The PCR results for the R1 region of the Ell promoter are presented. Abbreviation: C, control MCF7 cells; OE, Ell3-overexpressing cells; siNS, non-specific siRNA; siER, siRNA targeting ER(α); siEll3, siRNA targeting Ell3. The error bars represent the standard errors from three independent experiments, which were each performed using triplicate samples. *P < 0.05 (Student's *t*-test).

We next assessed whether ER(α) directly binds to the *IL-20* promoter by chromatin immunoprecipitation (ChIP) analysis. We scanned the promoter region (< 1 kb) of human *IL-20* using a genome browser based on the ER(α) Chromatin Interaction Analysis with Paired-End Tag Sequencing (ChIA-PET) signal and then divided the *IL-20* promoter into four regions, R1 to R4 (Figure 3A, Left panel). We next performed ChIP analysis of MCF7 cells using an anti-ER(α) antibody. Normal rabbit IgG was used as a negative control, and anti-acetylated histone H3 (AcH3) was used as a positive control. PCR amplification revealed that the amount of immunoprecipitated R1 and R2 was increased by at least three-fold in Ell3 OE cells compared with that in the control MCF7 cells (Figure 3A, Right panel). To further assess whether ER(α) directly regulates the activity of the *IL-20* promoter, we next performed a luciferase activity assay with the *IL-20* promoter. As shown in Figure 3B, cotransfection of ER(α) with a luciferase reporter plasmid carrying the *IL-20* promoter increased the luciferase activity up to 3-fold, and the addition of Ell3 further increased the luciferase activity. To mutate the ER(α) binding site of the *IL-20* promoter, we scanned the estrogen response element sequences (ERE: 5′-AGGTCANNNTGACCT-3′) in the *IL-20* promoter (Supplementary Figure 1A). We identified two ERE half sites from -92 to -97 and -788 to -793 in the *IL-20* promoter (Supplementary Figure 1B). We mutated two of the ERE half sites of *IL-20* and then compared their luciferase activity with that of wild type upon ER(α) expression. As expected, ER(α)-mediated activation of the *IL-20* promoter with a point mutation at the ER half site was significantly lower than that of the wild type promoter (Figure 3C). When both of the ERE half sites in the *IL-20* promoter were mutated, the promoter activity was even lower than that of the promoter with a single mutation (Figure 3C). These results indicated that ER(α) is a transcriptional activator of the *IL-20* promoter. Since transfection of siER(α) into MCF7 cells resulted in the suppression of Ell3 protein and ER(α) protein levels (Figure 1B), we next examined whether ER(α) also regulates the transcription of Ell3. As expected, the siRNA-mediated suppression of ER(α) resulted in a decrease in the Ell3 transcript level (Figure 3D). To determine whether ER(α) directly regulates Ell3 expression, we scanned the Ell3 promoter (< 1 kb) and identified three EREs upstream of the Ell3 transcriptional start site (Figure 3E, left panel). We conducted ChIP assays to assess the binding of ER(α) to this potential binding site in the Ell3 promoter and confirmed that ER(α) bound to the R1 region of this promoter (Figure 3E, Right panel). Based on these results, we concluded that ER(α) activates the transcription of *IL-20* and Ell3, possibly by direct binding to the promoter of each gene.

### ER(α), GATA3 and FOXA1 collectively regulate IL-20 expression

GATA3 and FOXA1 are considered ER-related genes because they are co-expressed with ER(α) in breast tumors and in breast cancer cell lines [11]. In addition, these triple factors are components of an enhanceosome that functions in combinatorial control of the transcriptional network in ER(+) breast cancer cells [12].

**Figure 4:**
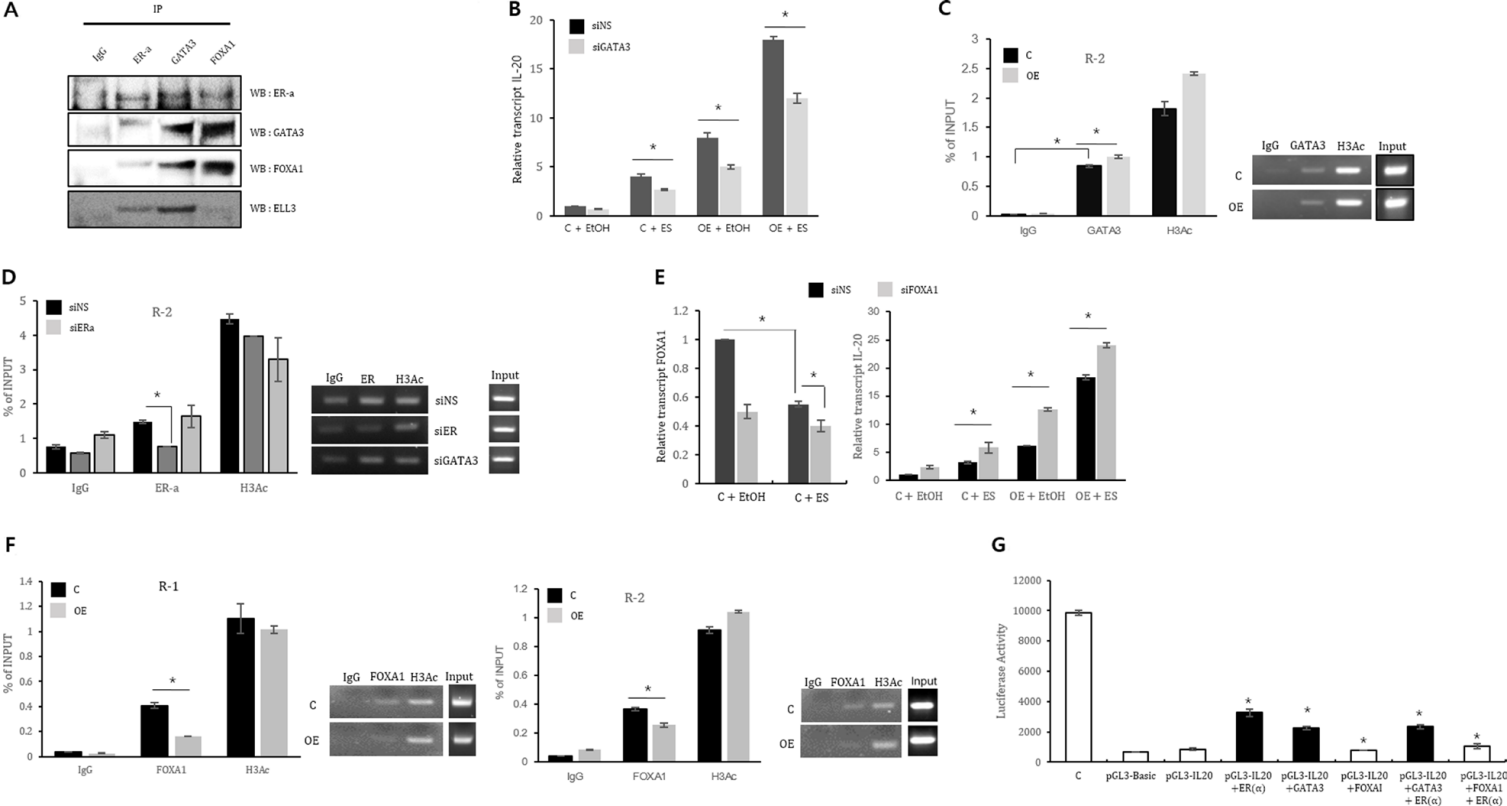
ER(α), GATA3 and FOXA1 collectively regulate *IL-20* expression (**A**) Co-immunoprecipitation of ER(α), GATA3, FOXA1 and Ell3. MCF7 cell lysates were precipitated with anti-ER(α), anti-GATA3 and anti-FOXA1-conjugated agarose and blotted with the indicated antibodies. (**B**) The effect of GATA3 suppression on *IL-20* expression in control and Ell3 OE cells in the presence or absence of estrogen. Control and Ell3 OE cells were transfected with siNS or siGATA3 for 24 h before estrogen treatment. At 24 h after estrogen treatment, *IL-20* expression was analyzed by real-time RT-PCR. (**C**) Chromatin from control and Ell3 OE cells was immunoprecipitated (ChIP) with antibodies against IgG, GATA3 and H3Ac. The PCR results for R1 and R2 of the *IL-20* promoter are presented. (**D**) Chromatin from MCF7 cells transfected with siNS, siER(α), and siGATA3 was immunoprecipitated (ChIP) with antibodies against IgG, ER(α) and H3Ac. The PCR results for R2 in the *IL-20* promoter are presented. (**E**) The effect of suppression of FOXA1 on *IL-20* expression in control and Ell3 OE cells. Control and Ell3 OE cells were transfected with siNS or siFOXA1 at 24 h before estrogen treatment. At 24 h after estrogen treatment, *IL-20* expression was analyzed by real-time RT-PCR. (**F**) Chromatin from control and Ell3 OE cells was immunoprecipitated (ChIP) with antibodies against IgG, FOXA1 and H3Ac. The PCR results for R1 and R2 in the *IL-20* promoter are presented. (**G**) Luciferase activity assays of the *IL-20* promoter in the presence of ER(α), GATA3 and FOXA1. 293T cells were transfected with the indicated plasmid for 24 h, and cell lysates were analyzed for luciferase activity. The positive control was the pGL3-control plasmid, and the negative control was the pGL3-Basic plasmid. Abbreviation: IP, immunoprecipitation; WB, western blot: siNS, non-specific siRNA; siGATA3, siRNA targeting GATA3; siER, siRNA targeting ER(α); siFOXA1, siRNA targeting FOXA1; C, control MCF7 cells; OE, Ell3-overexpressing cells; H3Ac, acetyl-histone H3. The error bars represent the standard errors from three independent experiments, which were each performed using triplicate samples. *P < 0.05 (Student's *t*-test).

Our results suggest that ER(α) is a transcriptional activator of *IL-20*; thus, we next evaluated whether GATA3 and FOXA1 are also related to *IL-20* expression. First, we assessed whether GATA3 and FOXA1 form a complex with ER(α) and Ell3. As expected, ER(α), GATA3 and FOXA1 co-immunoprecipitated with each other, indicating that these three proteins form a complex, at least in MCF7 cells (Figure 4A). Notably, Ell3 was associated with GATA3 but not with FOXA1 (Figure 4A).

We next examined whether GATA3 regulates *IL-20* expression by assessing the effect of siGATA3 on *IL-20* expression. As expected, GATA3 depletion resulted in the suppression of *IL-20* in control and Ell3 OE cells in both the presence and absence of estrogen (Figure 4B). We next examined whether GATA3 regulates *IL-20* expression by direct binding to the *IL-20* promoter. Because the results shown in Figure 4A revealed that ER(α) directly binds to GATA3, we hypothesized that the binding site of GATA3 overlaps with that of ER(α) in the *IL-20* promoter. To verify that GATA3 binds to these regions of the *IL-20* promoter, we performed ChIP analysis of MCF7 cells using an anti-GATA3 antibody. PCR amplification revealed that the amount of the R2 region of the *IL-20* promoter immunoprecipitated by the GATA3 antibody was increased by at least twentyfold compared to that immunoprecipitated by the IgG antibody (Figure 4C). In contrast to ER(α), which exhibited increased binding affinity to the *IL-20* promoter in Ell3 OE cells (Figure 3A), the binding of GATA3 to R2 was not enhanced in these cells, suggesting that Ell3 does not affect the binding affinity of GATA3 to the *IL-20* promoter (Figure 4C). To this end, we further examined the effect of GATA3 depletion on the binding affinity of ER(α) to this promoter. As shown in Figure 4D, the binding affinity of ER(α) to the *IL-20* promoter was not affected by the depletion of GATA3, despite the fact that GATA3 and ER(α) form a transcriptional complex and bind to the same region of this promoter.

In contrast to GATA3, the siRNA-mediated depletion of FOXA1 resulted in an increase in *IL-20* expression in both the presence and absence of estrogen, indicating that FOXA1 is a negative regulator of *IL-20* (Figure 4E). Notably, FOXA1 expression was significantly decreased in the presence of estrogen, suggesting that estrogen regulates its transcription (Figure 4E). In ChIP analysis, the amounts of the R1 and R2 regions of the *IL-20* promoter immunoprecipitated by the FOXA1 antibody were increased up to 10-fold compared to that immunoprecipitated by the IgG antibody (Figure 4F). In addition, the amounts of the R1 and R2 regions immunoprecipitated by the FOXA1 antibody was significantly lower in Ell3 OE cells than in control cells (Figure 4F).

Luciferase activity assays further confirmed that GATA3 enhances the activity of the *IL-20* promoter and that FOXA1 inhibits the enhancement of the activity of the *IL-20* promoter by GATA3 (Figure 4G). Thus, we conclude that GATA3 is a positive regulator and that FOXA1 is a negative regulator of *IL-20* in MCF7 cells.

## DISCUSSION

*IL-20* is a pleiotropic cytokine involved in cancer, atherosclerosis, rheumatoid arthritis, and stroke. *IL-20* has recently been reported to play a pivotal role in tumor progression, and its expression has been strongly associated with the clinical outcomes of breast cancer patients [13]. However, the transcriptional regulatory mechanism underlying adjustment of *IL-20* levels in cells is largely unknown. In this study, we showed that expression of the pro-inflammatory cytokine *IL-20* was regulated by a transcriptional complex composed of the transcription factors ER(α), GATA3, and FOXA1 and the transcriptional elongation factor Ell3 in ER(+) breast cancer cells.

**Figure 5:**
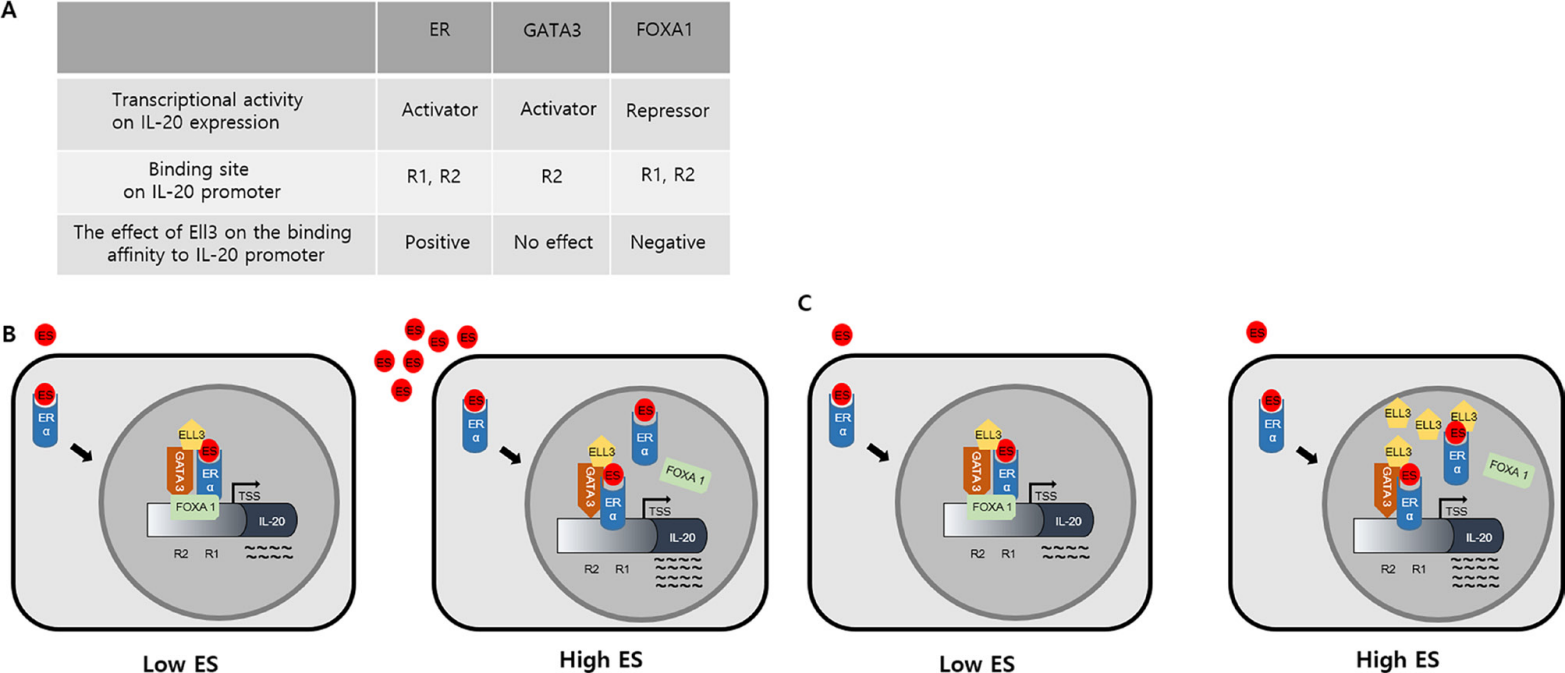
Proposed model of the effects of ER(α), GATA3, FOXA1 and Ell3 activities on *IL-20* expression in cancer cells (**A**) Summary of the effects of the transcriptional activities of ER(α), GATA3, FOXA1 and Ell3 on *IL-20* expression in MCF7 cells. (**B**) Effects of the estrogen-dependent transcriptional activities of ER(α), GATA3 and FOXA1 on *IL-20* expression. ER(α) and GATA3 are transcriptional activators, and FOXA1 is a transcriptional repressor of *IL-20* expression at low ES concentrations. At high ES concentrations, the binding affinity of ER(α) to the EREs is enhanced to prevent the binding of FOXA1, resulting in increased transcription of *IL-20*. (**C**) Effects of the Ell3-dependent transcriptional activities of ER(α), GATA3 and FOXA1 on *IL-20* expression. ER(α) and GATA3 are transcriptional activators, and FOXA1 is a transcriptional repressor of *IL-20* expression at a normal cellular concentration of Ell3. Ell3 overexpression enhances the binding affinity of ER(α) to the ERE to exclude FOXA1, resulting in increased transcription of *IL-20*.

As summarized in Figure 5A, we determined that FOXA1 is a transcriptional repressor, whereas ER(α) and GATA3 are transcriptional activators of *IL-20* expression. Our results revealed that the binding sites of these three factors overlap in the *IL-20* promoter. In addition, our results revealed the dynamics of the binding of ER(α) and FOXA1 to this promoter, which was dependent on the concentration of Ell3. Although Ell3 did not bind to FOXA1, as demonstrated by co-immunoprecipitation analysis (Figure 4A), Ell3 overexpression had a negative effect on the binding affinity of FOXA1 to the *IL-20* promoter. As FOXA1 and ER(α) compete for binding to the same regions of this promoter (R1 and R2), FOXA1 might be precluded from binding to this promoter by ER(α), the binding affinity of which was enhanced by Ell3. The fact that FOXA1 is a negative regulator of tumor promoting cytokine, *IL-20*, suggests that FOXA1 can be a useful prognostic marker for receptor-positive breast cancer.

Consistently, it was recently reported that FOXA1 expression is associated with a less aggressive phenotype and better prognosis in patients with hormone receptor-positive/HER2-negative breast cancer [14]. Because Ell3 is an additional factor to the triple complex of ER(α), GATA3 and FOXA1 and this complex fine-tunes the expression level of *IL-20*, it will be essential to assess the clinical meaning of the expression of FOXA1 in combination with the expression of Ell3 in ER(α) breast cancer patients.

Interestingly, ectopic expression of ER(α) induced the expression of *IL-20* in MDA-MB-231 cells. This finding suggests that the chromatin of the *IL-20* promoter in MDA-MB-231 cells is accessible to transcription factors. In other words, the lack of transcription factors is the one of main reasons for the low *IL-20* expression in MDA-MB-231 cells. However, *IL-20* expression was not significantly enhanced by estrogen treatment in ER(α)-transfected MDA-MB-231 cells, in contrast to MCF7 cells. It has been previously reported that the transfection of all three transcription factors, including ER(α), GATA3 and FOXA1, is necessary to restore the estrogen-responsive growth of ER(−) MDA-MB-231 cells [12]. It can be predicted that formation of a transcriptional complex consisting of at least Ell3 and GATA3 is required to restore the estrogen responsiveness of *IL-20* expression in MDA-MB-231 cells.

In summary, our findings have shown that *IL-20* expression in breast cancer is regulated by a transcriptional complex composed of transcription factors, including ER(α), GATA3, and FOXA1, as well as a transcriptional elongation factor, Ell3 (Figure 5). Recent evidence indicates that *IL-20* is a therapeutic target in various cancers, including breast cancer, and that an anti-*IL-20* monoclonal antibody has therapeutic potential for alleviating inflammation and osteolysis associated with tumor progression [9, 13, 15, 16]. Therefore, we expect that the results of our study will facilitate the development of a new therapeutic strategy for targeting *IL-20* expression in various diseases, including cancer.

## MATERIALS AND METHODS

### Cell culture

Ell3-overexpressing (Ell3 OE) and control MCF7 cells were cultured in DMEM containing 10% fetal bovine serum and 1% penicillin/streptomycin. The Ell3 OE cell line was constructed as described previously [6].

### Antibodies and immunoblot

The antibodies used in this study included anti-ER(α) (Cell Signaling, #8644), anti-Ell3 (Santa Cruz, Sc-242614), anti-ß-actin (Santa Cruz, Sc-47778), anti-GATA3 (Cell Signaling, #5852), and anti-FOXA1 (Cell Signaling, #58613). For immunoblot analysis, cells were lysed in tissue lysis buffer (20 mM Tris base, pH 7.4, 137 mM NaCl, 2 mM EDTA, 1% Triton X-100, 25 mM β-glycerophosphate, 2 mM sodium pyrophosphate, 10% glycerol, 1 mM sodium orthovanadate, 1 mM benzamidine, and 1 mM phenylmethylsulfonyl fluoride). Total cell extracts were resolved by sodium dodecyl sulfate-polyacrylamide gel electrophoresis (SDS–PAGE), transferred to Immobilon-P membranes (Millipore, Bedford, USA), and blotted with antibodies. Immunoreactivity was detected by enhanced chemiluminescence (ECL; Bio-Rad, CA, USA).

### Quantification of IL-20 in culture medium

A human *IL-20* immunoassay kit was used for *IL-20* quantification (DL200, R&D Systems). The optical density of each well was determined using a microplate reader at 450 nm.

### Transfection with siRNA

MCF7 cells were transfected with siRNA using siGENOME (Ell3, M-014601-01-0005; ER(α), L-003401-00-0010; GATA3, L-003781-00-0010; and FOXA1,L-010319-00-0010), which was provided by Dharmacon (distributed by ThermoScientific/AbGen, Epsom, UK). Cells were transfected with either target siRNA or non-specific siRNA using Lipofectamine 2000 (Invitrogen) in OPTI-MEM (Invitrogen) according to the manufacturer's instructions.

### Real-time reverse transcription PCR (Real-time RT-PCR)

RNA was isolated using TRI-reagent (Sigma-Aldrich, St. Louis, MO, USA). Total RNA (1 μg) was reverse transcribed using a 1st Strand cDNA Synthesis system (LeGene, San Diego, CA, USA) according to the manufacturer's protocol. Real-time PCR was performed in triplicate using the primers listed in Supplementary Table 1, with TOPreal qPCR 2X PreMIX (Enzynomics, Korea) and a CFX96 Real-time System (Bio-Rad Laboratories, Richmond, VA, USA). Expression levels were normalized to that of glyceraldehyde 3-phosphate dehydrogenase (GAPDH).

### Immunoprecipitation

Cells were washed with phosphate-buffered saline (PBS) and harvested in lysis buffer. Cell lysates were cleared by centrifugation at 12,000 rpm for 15 min at 4°C. For immunoprecipitation, the lysates were incubated with 4 μg of antibody for 16 h at 4°C, followed by incubation with 30 μl of protein G beads for 1 h at 4°C. The beads were washed once in lysis buffer. Then, the samples were boiled in SDS–PAGE sample buffer and analyzed by western blotting.

### Chromatin immunoprecipitation

A 1% formaldehyde solution was added to the cell culture medium for 10 min at 37°C. Cells were washed three times with cold PBS and then resuspended in lysis buffer (1% SDS, 10 mM EDTA, and 50 mM Tris-HCl, pH 8.1) with 1 mM phenylmethylsulfonyl fluoride (PMSF). After brief sonication, the lysates were cleared by centrifugation and diluted 5-fold with dilution buffer (0.01% SDS, 1% Triton X-100, 1.2 mM EDTA, 16.7 mM Tris-HCl, pH 8.1, and 167 mM NaCl) containing PMSF. Then, the lysates were incubated with an anti-ER(α) antibody overnight at 4°C. Immune complexes were precipitated with Protein A/G Plus Agarose. The precipitates were sequentially washed with low-salt wash buffer (0.1% SDS, 1% Triton X-100, 2 mM EDTA, 20 mM Tris-HCl, pH 8.1, and 150 mM NaCl), high-salt wash buffer (0.1% SDS, 1% Triton X-100, 2 mM EDTA, 20 mM Tris-HCl, pH 8.1, and 500 mM NaCl) and LiCl wash buffer (0.25 M LiCl, 1% NP-40, 1% deoxycholate, 1 mM EDTA, and 10 mM Tris-HCl, pH 8.1). After the final wash, elution buffer (1% SDS and 0.1 M NaHCO_3_) was added, followed by incubation at room temperature for 15 min with rotation. Then, the formaldehyde crosslinking was reversed by adding 0.3 M NaCl and heating at 65°C for 4 h. Next, proteinase K was added, followed by incubation at 45°C for 1 h. Then, DNA was recovered by phenol–chloroform extraction and ethanol precipitation. The resulting pellets were resuspended in TE buffer and subjected to PCR using primers for the promoter of *IL-20* or Ell3. The PCR products were separated by agarose gel electrophoresis.

### Luciferase reporter constructs and assay

The 5′ upstream regulatory region of the *IL-20* gene (−808 ~ + 1) was PCR amplified and cloned into the pGL3 luciferase reporter vector (Promega, Madison, WI, USA). Two potential ER(α) binding sites (ERE: estrogen response element) (−791~-792 and -93~-94) on the *IL-20* promoter were mutated as described in Supplementary Figure 1 and cloned into the pGL3 luciferase reporter vector. Then, 6 × 10^4^ cells of 293T were seeded in each well of a 24-well tissue plate and cotransfected with pGL3 reporter plasmid, pGL3-Basic plasmid, pGL3-control plasmid and SV40ß-galactosidase vector for normalizing transfection efficiency per well according to the manufacturer's instructions. Firefly luciferase activity was measured in cell lysates 24 h after transfection using the Luciferase Assay System (Promega). Experiments were repeated at least three times with three replicates per sample for each experiment. The results are normalized against ß-galactosidase activity.

### Statistical analysis

Each experiment was performed at least three times. Statistical significance of data analysis between two groups was determined using Student's *t*-test, and a *P value* of < 0.05 was considered significant. All statistical analyses were performed using the SAS statistical package, v.9.13 (SAS, Cary, NC, USA).

## SUPPLEMENTARY FIGURES AND TABLE


