# Supplementary material for: A transcriptional complex composed of ER(α), GATA3, FOXA1 and ELL3 regulates IL-20 expression in breast cancer cells
Source: Oncotarget. 2017 Apr 27;8(26):42752–60. doi: 10.18632/oncotarget.17459 (PMC5522103; doi:10.18632/oncotarget.17459)
Supplement: Supplementary file 1 [file oncotarget-08-42752-s001.pdf]

A transcriptional complex composed of ER(α), GATA3, FOXA1 and ELL3 regulates *IL-20* expression in breast cancer cells

Supplementary Materials

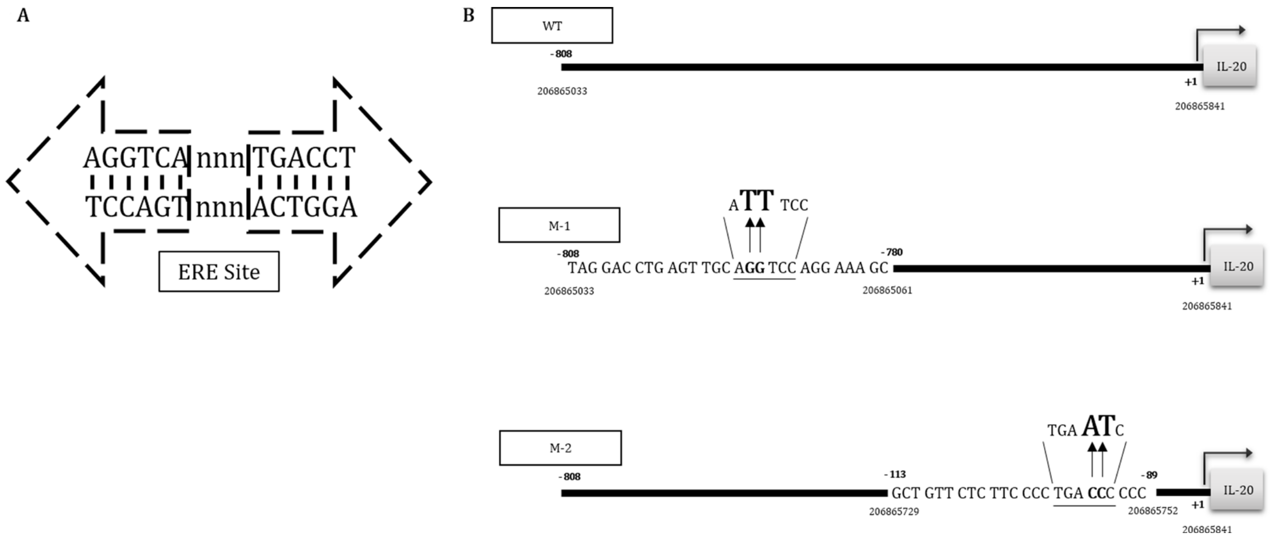

Supplementary Figure 1: Point mutational luciferase activity analysis of potential ER(α) binding sites. (**A**) The sequences of the estrogen response elements. (**B**) WT, IL-20 promoter region cloned into the pGL3 luciferase reporter vector. M-1, the GG sequences of the ERE half sites in the -788 ~ -793 region were mutated to TT sequences. M-2, the CC sequences of the ERE half sites in the -92 ~ -97 region were mutated to AT sequences.

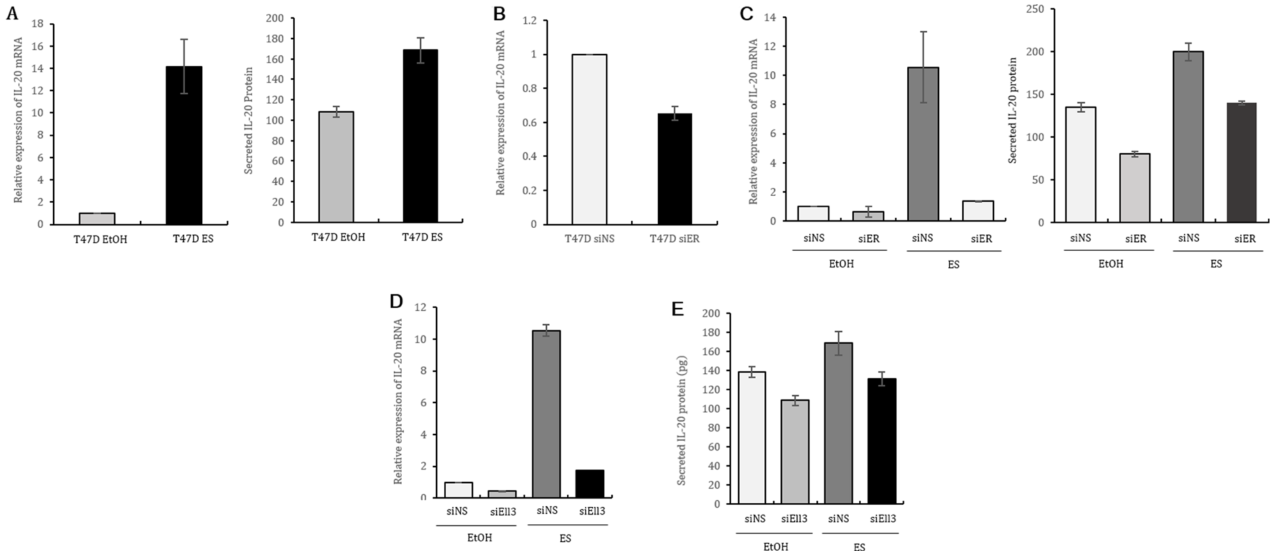

Supplementary Figure 2: Regulation of IL-20 expression by estrogen and ER(α) in the breast cancer cell line T47D. (**A**) The effect of estrogen treatment on *IL-20* expression in T47D cells. (**B**) The effect of siER(α) transfection on *IL-20* expression. (**C**) The effect of estrogen treatment on *IL-20* expression in T47D cells transfected with siNS or siER(α). (**D**) The effect of estrogen treatment on the expression of *IL-20* mRNA in T47D cells transfected with siNS or siEll3. (**E**) The effect of estrogen treatment on the expression of secreted *IL-20* protein in T47D cells transfected with siNS or siEll3. Abbreviations: EtOH, ethanol (0.1%); ES, estrogen (10 nM); siNS, non-specific siRNA; siER, siRNA targeting ER(α).

Supplementary Table 1: Primers used in this study

Gene Name		Sequences (5′ to 3′)
hIL-20	F (Forward)	CGC CAA TTC CTT TCT TAC CA
	R (Reverse)	CTA GTT CCC CCA AAG CCT TC
hER-a	F (Forward)	TAC TAC CTG GAG AAC GAG CC
	R (Reverse)	TGG TGG CTG GAC ACA TAT AG
hGATA3	F (Forward)	GCG GGC TCT ATC ACA AAA TGA
	R (Reverse)	GCC TTC GCT TGG GCT TAA T
hFOXA1	F (Forward)	AGG GCT GGA TGG TTG TAT TG
	R (Reverse)	GCC TGA GTT CAT GTT GCT GA
GAPDH	F (Forward)	ACC CAG AAG ACT GTG GAT GG
	R (Reverse)	TCT AGA CGG CAG GTC AGG TC
hELL3 Chip R1	F (Forward)	ACC AGC GTC TGA CCT CTC AC
	R (Reverse)	TTG CAG GTT CAG CTT TCA CTT
hELL3 Chip R2	F (Forward)	AGA GAC CAG AGG ATG GCT GA
	R (Reverse)	TGA CAG CCA ACT TTC ACT GC
hELL3 Chip R3	F (Forward)	AAT CCC CGG GAA CCT TAA C
	R (Reverse)	GCA GTG AGC GGA GAT CGT
hIL-20 Chip R1	F (Forward)	TCA CCA TTT TGG GGT TAA GAA
	R (Reverse)	CAT TGA TGT GGG TCA TTC CA
hIL-20 Chip R2	F (Forward)	TCA GTG CTG GGT AAA CTG GA
	R (Reverse)	TGC CAG GCA ATA TGC TAG AC
hIL-20 Chip R3	F (Forward)	TCA GTG CTG TGC CAA GCT AT
	R (Reverse)	AAT TTT GTT TGG GGC AGA AA
hIL-20 Chip R4	F (Forward)	CAT GGC ACT GTC GAA TTT TG
	R (Reverse)	GAG CAG GTT AGG GTG TTT GC
hIL-20 Promoter	F (Forward)	ATG CTCGAG CAT GGC ACT GTC GAA TTT
	R (Reverse)	ATG AGATCT TCC AGT GGA AGG AGT CCA
hIL-20 Point Mutation R1	F (Forward)	ATG CTCGAG CAT GGC ACT GTC GAA TTT
	R (Reverse)	GGG GAT TCA GGG GAA GAG ACA GCC
hIL-20 Point Mutation R2	F (Forward)	GCT GTT CTC TTC CCC TGA ATC CCC
	R (Reverse)	ATG AGA TCT TCC AGT GGA AGG AGT CCA
hIL-20 Point Mutation R3	F (Forward)	ATG CTC GAG CAT GGC ACT GTC GAA TTT
	R (Reverse)	CCT GGA AAT GCA ACT CAG GTC CTA GAC
hIL-20 Point Mutation R4	F (Forward)	TAG GAC CTG AGT TGC ATT TCC AGG AAA
	R (Reverse)	ATG AGATCT TCC AGT GGA AGG AGT CCA
